# Supplementary material for: Emergent quasiparticles at Luttinger surfaces
Source: Nat Commun. 2022 Mar 23;13:1561. doi: 10.1038/s41467-022-29190-y (PMC8943186; doi:10.1038/s41467-022-29190-y)
Supplement: Supplementary file 1 — Supplementary Information [file 41467_2022_29190_MOESM1_ESM.pdf]

Supplementary Note 1

In these Supplementary Notes we present a proof of Luttinger's theorem [1] somehow complementary to existing ones [1–5], with the purpose of clarifying under which conditions the theorem is valid, as recently discussed in great detail by Ref. [6].

S1. LUTTINGER'S THEOREM

The first step of Luttinger's theorem is the trivial equivalence

$$\begin{aligned} N &= \sum_{\mathbf{k}\sigma} T \sum_n \mathrm{e}^{i\epsilon_n \eta}\, G(i\epsilon_n, \mathbf{k}) = V + \sum_{\mathbf{k}\sigma} T \sum_n G(i\epsilon_n, \mathbf{k}) \\ &= V + \sum_{\mathbf{k}\sigma} T \sum_{i\epsilon_n} \frac{\partial}{\partial i\epsilon_n} \ln G(i\epsilon_n, \mathbf{k})\Big) + T \sum_{i\epsilon_n} G(i\epsilon_n, \mathbf{k}) \, \frac{\partial \Sigma(i\epsilon_n, \mathbf{k})}{\partial i\epsilon_n} \,, \end{aligned} \tag{S1.1}$$

where $\eta > 0$ is infinitesimal, N is the number of electrons, V the number of sites, $G(i\epsilon_n, \mathbf{k})$ and $\Sigma(i\epsilon_n, \mathbf{k})$ the spin-independent Green's function and self-energy in momentum $\mathbf{k}$ and Matsubara frequency $\epsilon_n = (2n+1)\pi T$.

Now, consider the Luttinger Ward functional $\Phi[G]$ [7, 8], and assume to shift the frequencies of all internal Green's functions by the same amount $i\omega = i\,2\pi T$. Evidently, $\Phi[G]$ does not change under such shift, thus

$$\begin{aligned} \delta\Phi[G] = 0 &= \sum_{\mathbf{k}\sigma} T \sum_n \mathrm{e}^{i\epsilon_n \eta}\, \Sigma(i\epsilon_n, \mathbf{k}) \Big(G(i\epsilon_n + i\omega, \mathbf{k}) - G(i\epsilon_n - i\omega, \mathbf{k}) \Big) \\ &= - \sum_{\mathbf{k}\sigma} T \sum_n \mathrm{e}^{i\epsilon_n \eta}\, G(i\epsilon_n, \mathbf{k}) \Big(\Sigma(i\epsilon_n + i\omega, \mathbf{k}) - \Sigma(i\epsilon_n - i\omega, \mathbf{k}) \Big) \,. \end{aligned} \tag{S1.2}$$

We emphasise that the equivalence between the two sums in Eq. (S1.2) is a trivial consequence of the summation over all Matsubara frequencies $\epsilon_n = (2n+1)\,\pi T$, $n = [-\infty, \infty]$ and of the fact that the series decays faster than $1/\epsilon_n$, which implies that we can safely set $\eta = 0$ before performing the summation over n, and thus that no boundary term appears.
Luttinger's theorem is valid if, upon defining

$$\begin{aligned} I &\equiv \frac{\delta\Phi[G]}{2i\omega} = \sum_{\mathbf{k}\sigma} T \sum_n G(i\epsilon_n, \mathbf{k}) \, \frac{\Sigma(i\epsilon_n + i\omega, \mathbf{k}) - \Sigma(i\epsilon_n - i\omega, \mathbf{k})}{2i\omega} = 0 \,, \\ I_L &\equiv \sum_{\mathbf{k}\sigma} T \sum_n G(i\epsilon_n, \mathbf{k}) \, \frac{\partial \Sigma(i\epsilon_n, \mathbf{k})}{\partial i\epsilon_n} \,, \end{aligned} \tag{S1.3}$$

the following equivalence holds for $\omega = 2\pi T \to 0$, thus for $T \to 0$,

$$0 = I \stackrel{?}{=} I_L \,, \tag{S1.4}$$

in which case we are allowed to drop the last term on the right hand side of Eq. (S1.1), and thus recover Luttinger's standard expression of the number of particles.

The equivalence (S1.4) might seem obvious, but in reality is not so. We first note that, since $G(i\epsilon, \mathbf{k})$ and $\Sigma(i\epsilon, \mathbf{k})$ have discontinuous imaginary parts at $\epsilon = 0$, the summation I must be dealt with care in the $T \to 0$ limit, since the functions that are summed may be on different sides of the imaginary axis. Therefore, we can write

$$\begin{aligned} I = \sum_{\mathbf{k}\sigma} \Bigg\{ T \sum_{n\geq 1 \vee n\leq -2} G(i\epsilon_n, \mathbf{k}) \, \frac{\Sigma(i\epsilon_n + i\omega, \mathbf{k}) - \Sigma(i\epsilon_n - i\omega, \mathbf{k})}{2i\omega} \\ + \frac{1}{4\pi i} \, G(i\pi T, \mathbf{k}) \Big(\Sigma(3i\pi T, \mathbf{k}) - \Sigma(-i\pi T, \mathbf{k}) \Big) \\ + \frac{1}{4\pi i} \, G(-i\pi T, \mathbf{k}) \Big(\Sigma(i\pi T, \mathbf{k}) - \Sigma(-3i\pi T, \mathbf{k}) \Big) \Bigg\} \,, \end{aligned} \tag{S1.5}$$

so that the two summations $n \geq 1$ and $n \leq -2$ only involve functions on the same side of the imaginary axis. At this stage, it is tempting to straight take the $T \to 0$ limit and conclude that

$$\begin{aligned} I \xrightarrow[T\to 0]{} \sum_{\mathbf{k}\sigma} \Bigg\{ \int_{0^+}^{\infty} \frac{d\epsilon}{2\pi} \, G(i\epsilon, \mathbf{k}) \, \frac{\partial \Sigma(i\epsilon, \mathbf{k})}{\partial i\epsilon} + \int_{-\infty}^{0^-} \frac{d\epsilon}{2\pi} \, G(i\epsilon, \mathbf{k}) \, \frac{\partial \Sigma(i\epsilon, \mathbf{k})}{\partial i\epsilon} \\ + \frac{1}{\pi} \, \mathrm{Re}\, G(i0^+, \mathbf{k}) \, \mathrm{Im}\, \Sigma(i0^+, \mathbf{k}) \Bigg\} \,, \end{aligned} \tag{S1.6}$$

which is correct to leading order in T. On the contrary, the $T \to 0$ limit of I_L does not pose any problem, and reads

$$I_L \xrightarrow[T\to 0]{} \sum_{\mathbf{k}\sigma} \Bigg\{ \int_{0^+}^{\infty} \frac{d\epsilon}{2\pi} \, G(i\epsilon, \mathbf{k}) \, \frac{\partial \Sigma(i\epsilon, \mathbf{k})}{\partial i\epsilon} + \int_{-\infty}^{0^-} \frac{d\epsilon}{2\pi} \, G(i\epsilon, \mathbf{k}) \, \frac{\partial \Sigma(i\epsilon, \mathbf{k})}{\partial i\epsilon} \Bigg\} \,. \tag{S1.7}$$

Since $I = 0$, it follows that

$$I_L = -\frac{1}{\pi} \sum_{\mathbf{k}\sigma} \mathrm{Re}\, G(i0^+, \mathbf{k}) \, \mathrm{Im}\, \Sigma(i0^+, \mathbf{k}) \,, \tag{S1.8}$$

is just a boundary term on the imaginary frequency axis. We emphasise that Eq. (S1.8) is just the leading order in T, hence higher order terms are neglected.

If those terms can be indeed neglected, Eq. (S1.8) implies that $I_L = 0$ if $\mathrm{Re}\,G_+(\epsilon,\mathbf{k})\,\mathrm{Im}\,\Sigma_+(\epsilon,\mathbf{k})$ is smooth around $\epsilon = 0$ and

$$\frac{1}{\pi}\lim_{\epsilon\to 0}\mathrm{Re}\,G_+(\epsilon,\mathbf{k})\,\mathrm{Im}\,\Sigma_+(\epsilon,\mathbf{k}) = -\lim_{\epsilon\to 0}\Xi(\epsilon,\mathbf{k})\,A_{\mathrm{qp}}(\epsilon,\mathbf{k}) = 0\,, \tag{S1.9}$$

where, by definition,

$$\Xi(\epsilon,\mathbf{k}) = -Z(\epsilon,\mathbf{k})\,\mathrm{Re}\,G_+(\epsilon,\mathbf{k})^{-1}\,,\quad A_{\mathrm{qp}}(\epsilon,\mathbf{k}) = -\frac{1}{\pi}\,\frac{\mathrm{Im}\,G_+(\epsilon,\mathbf{k})}{Z(\epsilon,\mathbf{k})}\,. \tag{S1.10}$$

Eq. (S1.9) does holds under the analytic assumptions we make in the article and that lead to

$$\lim_{\epsilon\to 0}A_{\mathrm{qp}}(\epsilon,\mathbf{k}) = \lim_{\epsilon\to 0}\delta\big(\epsilon - \Xi(\epsilon,\mathbf{k})\big)\,. \tag{S1.11}$$

Therefore, those same assumptions seem to imply that $I_L = 0$ at leading order in T, and thus the conventional Luttinger's theorem applies.

There is however a caveat that has been recently highlighted in Ref. [6]. Imagine that

$$\sum_{\mathbf{k}\sigma} G(i\epsilon,\mathbf{k})\,\frac{\partial\Sigma(i\epsilon,\mathbf{k})}{\partial i\epsilon} = k\,\frac{\partial\ln\Omega(i\epsilon)}{\partial i\epsilon} + R(i\epsilon)\,, \tag{S1.12}$$

with integer k, which ensures regular analytic properties, and $R(i\epsilon)$ such that its integral over ϵ vanishes identically. Assume further that $\Omega(i\epsilon) = \Omega(-i\epsilon)^*$ goes to 1 for $\epsilon \to \pm\infty$ faster than $1/\epsilon$, and $\mathrm{Im}\,\Omega_+(\epsilon)$ vanishes linearly as $\epsilon \to 0$ whereas $\mathrm{Re}\,\Omega_+(\epsilon \to 0) = \Omega_+(0) \neq 0$. Under the above assumptions,

$$\begin{aligned} I_L &= k\int_{-\infty}^{\infty}\frac{d\epsilon}{2\pi}\,\frac{\partial\ln\Omega(i\epsilon)}{\partial i\epsilon} = -\frac{k}{2\pi i}\Big(\ln\Omega(i0^+) - \ln\Omega(i0^-)\Big) \\ &= -\frac{k}{\pi}\,\tan^{-1}\frac{\mathrm{Im}\,\Omega(i0^+)}{\mathrm{Re}\,\Omega(i0^+)} \in \mathbb{Z}\,, \end{aligned} \tag{S1.13}$$

in which case the Luttinger integral would be quantised in integers. We note that the above result is actually not incompatible with Eq. (S1.8) being zero. Indeed, since $\mathrm{Im}\,\Omega(i\epsilon) \propto i\epsilon$ for $\epsilon \to 0$, then

$$\mathrm{Im}\,\ln\Omega(i\pi T) = \mathrm{Im}\,\ln\Big(\mathrm{Re}\,\Omega(i\pi T) + i\,\mathrm{Im}\,\Omega(i\pi T)\Big) \simeq \frac{\mathrm{Im}\,\Omega(i\pi T)}{\mathrm{Re}\,\Omega(i\pi T)} + O\big(T^3\big) \sim T\,, \tag{S1.14}$$

naïvely vanishes at leading order in T, despite the whole series may converge to $\pm\pi$. Similarly, Eq. (S1.8) is only the first term of a series expansions in powers of T. That series may indeed converge to a value different from the leading term.

One thus concludes [6] that

- the condition

$$\lim_{\epsilon\to 0} \mathrm{Re}\, G_+(\epsilon,\mathbf{k})\, \mathrm{Im}\, \Sigma_+(\epsilon,\mathbf{k}) = 0\ , \tag{S1.15}$$

which is fulfilled under the analytic assumptions we make in the work, only guarantees that the Luttinger integral I_L is quantised in integer values, namely that Luttinger's theorem generically leads to an estimate of the electron number wrong by an integer number.

S2. BEYOND CONVENTIONAL LUTTINGER'S THEOREM

Let us elaborate further on this point, still closely following Ref. [6]. Eq. (S1.1) at $T = 0$ is

$$\begin{aligned} N &= \sum_{\mathbf{k}\sigma} \left[\frac{1}{2} - \int_{-\infty}^{\infty} \frac{d\epsilon}{2\pi}\, \frac{\partial \ln G(i\epsilon,\mathbf{k})}{\partial i\epsilon} + \int_{-\infty}^{\infty} \frac{d\epsilon}{2\pi}\, G(i\epsilon,\mathbf{k})\, \frac{\partial \Sigma(i\epsilon,\mathbf{k})}{\partial i\epsilon} \right] \\ &= \sum_{\mathbf{k}\sigma} \left[\frac{1}{2} - \frac{\delta(\infty,\mathbf{k}) - \delta(0^+,\mathbf{k})}{\pi} + \int_{-\infty}^{\infty} \frac{d\epsilon}{2\pi}\, G(i\epsilon,\mathbf{k})\, \frac{\partial \Sigma(i\epsilon,\mathbf{k})}{\partial i\epsilon} \right] \\ &\equiv \sum_{\mathbf{k}} n_*(\mathbf{k})\,, \end{aligned} \tag{S2.1}$$

where

$$\delta(\epsilon,\mathbf{k}) = \arg\big(G(i\epsilon,\mathbf{k})\big)\,, \tag{S2.2}$$

and so

$$\delta(\infty,\mathbf{k}) = -\frac{\pi}{2}\,,\quad \delta(0^+,\mathbf{k}) = \begin{cases} -\pi & E(\mathbf{k}) > 0\,, \\ -\frac{\pi}{2} & E(\mathbf{k}) = 0\,, \\ 0 & E(\mathbf{k}) < 0\,. \end{cases} \tag{S2.3}$$

In the perturbative regime, where Landau's adiabatic hypothesis is valid, the Luttinger integral

$$I_L \equiv \sum_{\mathbf{k}\sigma} \int_{-\infty}^{\infty} \frac{d\epsilon}{2\pi}\, G(i\epsilon,\mathbf{k})\, \frac{\partial \Sigma(i\epsilon,\mathbf{k})}{\partial i\epsilon} = 0\,, \tag{S2.4}$$

so that

$$n_*(\mathbf{k}) = \begin{cases} 0 & E(\mathbf{k}) > 0\,, \\ 1 & E(\mathbf{k}) = 0\,, \\ 2 & E(\mathbf{k}) < 0\,, \end{cases} \tag{S2.5}$$

which is the standard expression of Luttinger's theorem. The point at which perturbation theory breaks down is also that at which a Luttinger surface first emerges. Suppose that $\mathbf{k}$ lies on that surface. It follows that right before the breakdown (b.b.)

$$n_*(\mathbf{k}) = 2\theta\big(- E(\mathbf{k})\big) = 2\theta\Big(\mathrm{Re}\, G_{\text{b.b.}}(0,\mathbf{k})\Big)\,. \tag{S2.6}$$

Assuming that conventional Luttinger's theorem holds true even at the breakdown point, we must conclude that $n_*(\mathbf{k}) = 1$, since $\delta(0^+,\mathbf{k}) = -\pi/2$ when $\mathrm{Re}\, G(0,\mathbf{k}) = 0$, namely when a double zero of $\mathrm{Re}\, G(i\epsilon,\mathbf{k}) = \mathrm{Re}\, G(-i\epsilon,\mathbf{k})$ appears right at $\epsilon = 0$. After the breakdown (a.b.), that double zero generically splits into two, one zero moving along the $\epsilon > 0$ semi axis, and the other symmetrically along $\epsilon < 0$. It follows that after the breakdown $\mathrm{Re}\, G_{\text{a.b.}}(0,\mathbf{k})$ has the opposite sign of $\mathrm{Re}\, G_{\text{b.b.}}(0,\mathbf{k})$ before the breakdown. Should the breakdown occur simultaneously at all momenta $\mathbf{k}$, it would correspond to a Mott transition, beyond which $n_*(\mathbf{k})$ remains pinned at one, i.e., half-filled density. What does it happen when instead the breakdown just gives rise to a Luttinger surface with a single-particle pseudo gap rather than a hard one? According to Ref. [6] also there $n_*(\mathbf{k})$ remains pinned at one, which would imply that, in absence of additional Fermi pockets, the system should be incompressible despite the existence of 'quasiparticles'. Correspondingly, doping away from half-filling necessarily leads to the emergence of Fermi pockets, contributing to two electrons per $\mathbf{k}$-point when they are electron-like, and zero when they are hole-like.
Therefore, one can write for a generic $\mathbf{k}$ and just after perturbation theory has broken down

$$n_*(\mathbf{k}) = \theta\Big(\mathrm{Re}\, G_{\text{a.b.}}(0,\mathbf{k})\Big) + \theta\Big(\mathrm{Re}\, G_{\text{b.b.}}(0,\mathbf{k})\Big)\,, \tag{S2.7}$$

which remains the conventional expression (S2.6) for all $\mathbf{k}$'s not affected by the breakdown, i.e., where $\mathrm{sign}\Big(\mathrm{Re}\, G_{\text{a.b.}}(0,\mathbf{k})\Big) = \mathrm{sign}\Big(\mathrm{Re}\, G_{\text{b.b.}}(0,\mathbf{k})\Big)$, while it is pinned to value 1 if $\mathrm{sign}\Big(\mathrm{Re}\, G_{\text{a.b.}}(0,\mathbf{k})\Big) = -\mathrm{sign}\Big(\mathrm{Re}\, G_{\text{b.b.}}(0,\mathbf{k})\Big)$.
Now, let us assume without proof that in the perturbative regime the volume of the interacting Fermi surface is the same as in the Hartree-Fock approximation. If so, then

$$N = \sum_{\mathbf{k}\sigma} \theta\big(- E(\mathbf{k})\big) = \sum_{\mathbf{k}\sigma} \theta\Big(\mathrm{Re}\, G_{\text{b.b.}}(0,\mathbf{k})\Big) = \sum_{\mathbf{k}\sigma} \theta\big(- \epsilon(\mathbf{k})\big)\,, \tag{S2.8}$$

where, as in the work, we have absorbed the Hartree-Fock self-energy $\Sigma_{HF}(\mathbf{k})$ into $\epsilon(\mathbf{k})$. It follows that in the non perturbative regime and through Eq. (S2.7),

$$\begin{aligned} N &= \sum_{\mathbf{k}} \theta\Big(\text{Re}\, G_{\text{a.b.}}(0,\mathbf{k})\Big) + \sum_{\mathbf{k}\sigma} \theta\big(-\epsilon(\mathbf{k})\big) \\ &= \frac{1}{2} \sum_{\mathbf{k}\sigma} \Big(\theta\big(-E(\mathbf{k})\big) + \theta\big(-\epsilon(\mathbf{k})\big)\Big) \equiv \sum_{\mathbf{k}} n_*(\mathbf{k})\,, \end{aligned} \tag{S2.9}$$

which is the formula that use in the work. We observe that $n_*(\mathbf{k})$ in the generalised expression (S2.9) differs from the conventional one $2\theta\big(-E(\mathbf{k})\big)$ by an integer, either positive or negative, in agreement with the previous discussion.

Remarkably, the simple formulas (S2.7) and (S2.9), despite relying on the unproven assumption (S2.8), has been shown [6] to reproduce the correct electron number in well known examples [9, 10] of compressible systems that violate conventional Luttinger's theorem.

---

[1] J. M. Luttinger, Phys. Rev. **119**, 1153 (1960), URL `https://link.aps.org/doi/10.1103/PhysRev.119.1153`.

[2] I. Dzyaloshinskii, Phys. Rev. B **68**, 085113 (2003), URL `https://link.aps.org/doi/10.1103/PhysRevB.68.085113`.

[3] K. Seki and S. Yunoki, Phys. Rev. B **96**, 085124 (2017), URL `https://link.aps.org/doi/10.1103/PhysRevB.96.085124`.

[4] M. Oshikawa, Phys. Rev. Lett. **84**, 3370 (2000), URL `https://link.aps.org/doi/10.1103/PhysRevLett.84.3370`.

[5] J. T. Heath and K. S. Bedell, New Journal of Physics **22**, 063011 (2020), URL `https://doi.org/10.1088%2F1367-2630%2Fab890e`.

[6] J. Skolimowski and M. Fabrizio (2021), unpublished.

[7] J. M. Luttinger and J. C. Ward, Phys. Rev. **118**, 1417 (1960), URL `https://link.aps.org/doi/10.1103/PhysRev.118.1417`.

[8] M. Potthoff, Condens. Mat. Phys. **9**, 557 (2006), URL `http://www.icmp.lviv.ua/journal/zbirnyk.47/011/abstract.html`.

[9] B. L. Altshuler, A. V. Chubukov, A. Dashevskii, A. M. Finkel'stein, and D. K. Morr, Europhysics Letters (EPL) **41**, 401 (1998), URL `https://doi.org/10.1209/epl/i1998-00164-y`.

[10] O. J. Curtin, Y. Nishikawa, A. C. Hewson, and D. J. G. Crow, Journal of Physics Communications **2**, 031001 (2018), URL `https://doi.org/10.1088/2399-6528/aab00e`.
